# Selecting Controls for Assessing Interaction in Nested Case-control Studies

**DOI:** 10.2188/jea.13.193

**Published:** 2007-11-30

**Authors:** John Cologne, Bryan Langholz

**Affiliations:** 1 Department of Statistics, Radiation Effects Research Foundation.; 2 Division of Biostatistics, Department of Preventive Medicine, University of Southern California Keck School of Medicine.

**Keywords:** nested case-control studies, matching, counter matching, models, statistical, atomic-bomb survivor studies

## Abstract

Background: Two methods for selecting controls in nested case-control studies — matching on *X* and counter matching on *X* — are compared when interest is in interaction between a risk factor *X* measured in the full cohort and another risk factor *Z* measured only in the case-control sample. This is important because matching provides efficiency gains relative to random sampling when *X* is uncommon and the interaction is positive (greater than multiplicative), whereas counter matching is generally efficient compared to random sampling.

Methods: Matching and counter matching were compared to each other and to random sampling of controls for dichotomous *X* and *Z*. Comparison was by simulation, using as an example a published study of radiation and other risk factors for breast cancer in the Japanese atomic-bomb survivors, and by asymptotic relative efficiency calculations for a wide range of parameters specifying the prevalence of *X* and *Z* as well as the levels of correlation and interaction between them. Focus was on analyses utilizing general models for the joint risk of *X* and *Z*.

Results: Counter-matching performed better than matching or random sampling in terms of efficiency for inference about interaction in the case of a rare risk factor *X* and uncorrelated risk factor *Z*. Further, more general, efficiency calculations demonstrated that counter-matching is generally efficient relative to matched case-control designs for studying interaction.

Conclusions: Because counter-matched designs may be analyzed using standard statistical methods and allow investigation of confounding of the effect of *X*, whereas matched designs require a non-standard approach when fitting general risk models and do not allow investigating the adjusted risk of *X*, it is concluded that counter-matching on *X* can be a superior alternative to matching on *X* in nested case-control studies of interaction when *X* is known at the time of case-control sampling.

With epidemiologic cohort studies, it is important that researchers utilize the most efficient nested case-control designs to study interaction between two risk factors, one of which is known for the entire cohort but the other of which can only be feasibly ascertained in a sample. The situation may be illustrated by the following real-life example. In the study of long-term radiation effects in the Japanese atomic-bomb Survivors — the so-called Life Span Study^1^ — case-control studies must be performed to investigate certain cancer risk factors because it may be impractical to obtain risk factor information for all 120,000 cohort members. Land et al.^2^ studied interaction between radiation and three other breast-cancer risk factors (age at first full-term pregnancy, number of births, and cumulative time of lactation) after matching on radiation dose in a case-control interview study. A major aim of the analysis was to investigate the scale of interaction using general risk models that differ from the standard log-linear model typically employed in case-control analyses.^3^^,^^4^

Matching on exposure can increase efficiency relative to random sampling of controls for the analysis of interaction when the exposure is rare and there is a positive (super-multiplicative) interaction.^5^ Thus, because radiation dose is available on all Life Span Study members and large doses are rare, matching on radiation dose might be considered a reasonable strategy. However, matching on radiation dose prevents studying the risk of radiation after adjusting for other risk factors measured in the case-control sample. Furthermore, when exploring interaction in general risk models such as excess relative risk (ERR) models,^6^ the analysis of matched data requires special methods. Such models typically require a parameter for the exposure main effect; if the case-control sample is matched on exposure, this parameter cannot be estimated from the case-control sample and must instead be substituted with the cohort estimate,^2^ which is not adjusted for the effects of the other factors.

Counter-matching is a method of control selection that can be thought of most simply as a way of reducing the frequency of concordant matched pairs in a pair-matched case-control study when surrogate information on exposure exists prior to sampling.^7^ Counter matching is not limited to matched-pair designs, though, and may be applied more generally to any number of controls and to unmatched case-control studies.^8^ It has been shown that, when exposure is known prior to sampling, counter-matching on exposure can increase efficiency relative to random sampling of controls for assessing interaction with, or confounding by, another factor to be measured in the case-control sample.^9^ In contrast to matching, counter-matching does not preclude estimating the confounder-adjusted exposure parameter from the case-control sample. Thus, counter-matching would be preferred for studies of interaction when general risk models are to be used unless there is a marked loss of efficiency compared to matching. An important question is therefore whether matching or counter-matching leads to greater statistical efficiency in the interaction analyses when using general risk models.

We therefore investigated the efficiency of matching and counter-matching for studying interaction in the context of the case-control study of breast cancer mentioned above, where controls are to be sampled from each risk set in such a way as to have similar age and birth year as the case. Although both matching and counter-matching are appropriate for this purpose and both can provide efficiency gains relative to random sampling, the efficiency gains with counter matching are more general than those with matching, and counter-matching and matching have not been directly compared under the circumstances where matching can result in increased statistical efficiency relative to random sampling. We therefore compared them to each other as well as to random (unmatched) control selection using a small simulation study and general large-sample efficiency calculations. Our results should provide guidance to researchers contemplating nested case-control designs in which interaction between two factors is an important study issue.

## METHODS

Let *X* represent exposure, which is known for all members of the cohort (e.g., radiation exposure in the Life Span Study). Let *Z* be any factor to be measured in the case-control sample, whose ascertainment requires considerable effort (e.g., any breast-cancer risk factor that requires assessment by interview).

### Matching and counter-matching

Matching. Matching on *X* involves selecting the specified number of controls at random from among all at-risk subjects with the same exposure status as the case. With exposure-matched data there is, by design, no variation in exposure status between the cases and controls, so that parameters related to the main effect of exposure cannot be estimated. In a model in which the main effects for *X* and *Z* are multiplicative (such as in equations 1 and 2 below), the variation in the relative risk associated with *Z* across the exposure-matched case-control sets provides sufficient information to estimate the risk of *Z* and the interaction parameter without estimating the risk of exposure *X*. However, the exposure-matched design precludes estimation of the exposure effect required in more general risk models (such as equations 3 and 4 below), so Land et al.^2^ proposed substituting the cohort estimate of the main effect of *X*. Because that estimate is not adjusted for possible confounding or effect modification by *Z*, they further proposed centering the factor *Z* at its mean in the case-control sample. However, if *Z* is a qualitative factor having a specific reference category, centering *Z* in the analysis leads to the estimation of parameters that do not directly represent the risks of the original levels of *Z*. Furthermore, simply substituting the cohort estimate ignores the effect of its uncertainty on the case-control analysis.

Counter-matching. Counter-matching is a relatively new method of sampling controls, which creates exposure diversity in the case-control data by exploiting information available on all members of the cohort. The design, analysis, and efficiency (relative to random sampling) of counter-matched studies are discussed elsewhere, so we refer the reader to those papers for a more detailed account.^9^^-^^13^ Briefly, controls are selected to increase the variation in *X* in the case-control set relative to random sampling, the opposite of the goal of matching.

To form the counter-matched sample for a continuous exposure *X*, such as radiation dose in the Life Span Study cohort, we would construct strata by calculating the (*m*+1) quantiles of the distribution of *X* among all cases in the cohort, where m is the number of controls to be sampled from each risk set (corresponding to a single case). For each risk set, one control would be sampled randomly from each of the non-case strata. Other factors *Z* would then be ascertained on the counter-matched sample. Of course case-control sets formed in this way cannot be analyzed as if they were randomly sampled, but sampling weights can be calculated and used in the analysis that adjust for the biased, stratified sampling. Each sampled subject has, as an associated weight, the number of qualified subjects in that exposure stratum in the cohort risk set. The case-control data therefore consist of a matched set identifier, the case-control status indicator, the (continuous) value of exposure *X*, the value(s) of the additional factor(s) *Z* measured only in the case-control sample, and the sampling weight for each subject. The analysis is then performed as in a standard matched case-control study, but with the addition of the log weights as an offset (a covariate with parameter fixed at 1).^14^ The continuous values of *X* are used in the analysis, rather than the categorical strata used for the counter matching.

### Models for the joint effect of radiation and other risk factors

For simplicity of illustration, in the present report we limit *X* to a dichotomous factor representing either significant radiation exposure (*X*=1 for dose > 1.0 Gray) or not (*X*=0) and Z to the dichotomous factor nulliparous (*Z*=1 for no full-term pregnancies) or not (*Z*=0). We use the term “interaction” to refer to statistical interaction in a model for the joint effect of two factors. This is what Rothman calls “effect-measure modification” to distinguish it from true biological interaction.^15^ The issue of statistical interaction in the risk of disease is complicated by the choice of scale for the main effects. Standard logistic regression programs most commonly use, by default, a log-linear scale so that the interaction model of the risk for factors *X* and *Z* has the form

$$R_{\text{L}}(x,z)=\exp(x\alpha_{\text{L}}+z\beta_{\text{L}}+xz\gamma_{\text{L}}),$$ (1)

where *x*, *z* are observed values of *X*, Z. Deviations from the main effects model, which assumes that the relative risk for the joint effect of *x* and *z* is the product exp{*xα*} × exp{*z**β*} of the marginal relative risks, are captured by the interaction term (xz*γ*_L_). This model is chosen mainly for reasons of convenience, because many statistical packages only support models of the log-linear form. However, depending on the specific study, other forms may be more appropriate, either to provide a more parsimonious description of the joint effect or to appropriately model hypothesized biological processes.^16^^,^^17^

Models for the joint effect of *X* and *Z* that are of particular interest in epidemiology and risk assessment include the multiplicative excess relative-risk (ERR) model

$$R_{\text{M}}(x,z)=[1+R_{X}(x,\alpha_{\text{M}})]\times [1+R_{Z}(z,\beta_{\text{M}})]\times S_{\text{M}}(x,z,\gamma_{\text{M}})$$ (2)

and the additive ERR model

$$R_{\text{A}}(x,z)=1+R_{\text{X}}(x,\alpha_{\text{A}})+R_{\text{Z}}(z,\beta_{\text{A}})+S_{\text{A}}(x,z,\gamma_{\text{A}}).$$ (3)

In equations 2 and 3, *R_X_*(*x*,*α*) and *R_Z_*(*z*,*β*) are excess relative-risk functions describing the marginal effects of *X* and *Z* based on parameters *α*_M_, *β*_M_ in the multiplicative model or *α*_A_, *β*_A_ in the additive model. The functions *S*_M_(*x*,*z*,*γ*_M_) and *S*_A_(*x*,*z*,*γ*_A_) are interaction terms on multiplicative or additive scale with parameters *γ*_M_ and *γ*_A_. When an interaction term is in the model, *R_X_*(*x*,*α*) or *R_Z_*(*z*,*β*) defines the excess relative risk when the other variable is equal to its null value.

Equations 2 and 3 without interaction terms are special cases of more general mixture models that can be written in various forms.^3^ To facilitate substituting the cohort estimate of the main effect of *X*, Land and associates^2^ used the form

$$R_{G}(x,z)=[1+R_{X}(x,\alpha_{\text{G}})]\times \{1+R_{Z}(z,\beta_{\text{G}})/[1+R_{X}(x,\alpha_{\text{G}}){]}^{\theta }\},$$ (4)

where *θ* = 0 corresponds to the pure (no interaction) multiplicative model and *θ* = 1 corresponds to the pure additive model. Another way of writing the mixture model is

$$R_{G}(x,z)=\{[1+R_{X}(x,\alpha_{\text{G}})]\times [1+R_{Z}(z,\beta_{\text{G}})]{\}}^{\tau }\{1+R_{X}(x,\alpha_{\text{G}})+R_{Z}(z,\beta_{\text{G}}){\}}^{1-\tau },$$ (5)

where *τ* = 1 corresponds to the pure multiplicative model and *τ* = 0 corresponds to the pure additive model. Equation 5 is more generally useful when the main effect of *X* can be estimated from the case-control data — e.g., when counter-matching is employed — and may be fit using the Epicure^©^ software (Hirosoft International Corp., Seattle, WA).

### Comparison of methods

For the simulation comparisons, we generated a hypothetical cohort of women based on the Life Span Study and results of the previous case-control study of Land et al.^2^ Substantial radiation exposure (dose > 1.0 Gray) and dichotomized parity (nulliparous) were each assigned a relative risk of 2.0 and their combined effect was taken to be slightly super-multiplicative with joint relative risk 2 × 2 × (8/7) = 4.57 (see Appendix). We then simulated 100 times the sampling and analysis of case-control sets from the cohort using each of the three sampling methods. A fixed number of controls (*m* = 1, 2, 3, 5, or 9) was sampled for each case without replacement from the simulated cohort, either matched on dichotomous radiation exposure status, counter-matched on exposure status, or sampled randomly without regard to exposure status (unmatched).

In the counter-matched design, we chose configurations in which there was balance in exposure status in each case-control set. Thus, when one control was selected it was sampled from subjects with the opposite exposure status as the case. When three, five, or nine controls were selected, two, three, or five controls were sampled from among the subjects with opposite exposure status as the case and one, two, or four from among subjects with the same exposure status as the case. Two controls results in an odd-sized case-control set; which strata will have an extra subject must be determined without regard to the exposure status of the case. We evaluated both approaches: 1) sampling so that each case-control set had two subjects in the unexposed stratum — i.e., one control was sampled from among the subjects with opposite exposure status as the case and one additional control was sampled from among the *unexposed* subjects — and 2) sampling so that each case-control set had two subjects in the exposed stratum — i.e., one control was sampled from among the subjects with opposite exposure status as the case and one additional control was sampled from among the *exposed* subjects. In the matched design, all controls were selected from the same exposure stratum as the case. With matching or counter-matching on exposure, all controls were sampled at random from within the appropriate stratum. With unmatched sampling, controls were sampled completely at random without regard to exposure stratum.

Comparison was in terms of inference about departure from pure multiplicative (equation 2) or additive (equation 3) ERR models. We studied 1) the power of the likelihood ratio test of interaction on additive and multiplicative scales, and 2) the mean and precision of estimates of the degree of interaction. Power was taken to be the proportion of simulations where the likelihood ratio test of no interaction was rejected at the 5% level. Because analyses using general ERR models with the matched design require inputting the cohort radiation ERR estimate but the other designs allow estimation of the radiation ERR from the case-control sample, it is not possible to directly compare interaction parameters between the matched analysis on the one hand and the counter-matched or unmatched analyses on the other. However, in the special case of either multiplicative model (equation 1 or 2), it is possible to test and estimate the degree of interaction between radiation and nulliparity in the exposure-matched design without the need for an exposure main-effect parameter. Thus, we compared the efficiencies of the various designs for estimating interaction on the multiplicative ERR scale (equation 2) with *S*_M_(*x*,*z*,*γ*_M_) = exp{*x**z**γ*_M_}. The variances of the interaction parameter estimates from the samples were compared to the full cohort variance (0.046), so a value as high as 1.0 means the sample is as efficient as the full cohort. All models were fit using the Epicure^®^ software.

To further compare these designs, we computed asymptotic relative efficiencies by calculating the expected variances of the estimated interaction term for each of the designs using analytic formulas^10^ with the same parameter values as those used in the simulations. These formulas are based on the log-linear model (equation 1), rather than the multiplicative ERR model (equation 2) used in the simulations, but the two models are equivalent because the interaction term in equation 1 is the logarithm of that in equation 2.

Because situations different from the breast-cancer study used here as an illustration may arise in practice, we further compared efficiencies of the three designs computed under more general scenarios (varying the prevalence of *X* and *Z* as well as the extent of interaction and association between them). The configurations studied were very similar to those used by Thomas and Greenland.^5^ Those configurations include all combinations of: 1) rare (prevalence=0.15), moderate (prevalence=0.5), or common (prevalence=0.85) *X*; 2) rare (prevalence=0.1), semi-moderate (prevalence=0.25), or moderate (prevalence=0.5) *Z*; 3) negative (OR=0.2), positive (OR=5.0), or no (OR=1.0) correlation between *X* and *Z*; and 4) joint effect of *X* and *Z* that is sub-multiplicative (exp{*γ*}=0.2), simple multiplicative (exp{*γ*}=1.0), or super-multiplicative (exp{*γ*}=5.0). The main effects rate ratios for both *X* and *Z* used in these calculations were exp{*α*} = exp{*β*} = 5.0. The ratios of asymptotic relative efficiencies of counter matching relative to either the matched or unmatched design were computed; thus, a value larger than 1.0 means counter-matching has greater efficiency.

## RESULTS

### Tests of model scale

Because the variance of a binomial proportion is a maximum when the proportion is 0.5, all of the power results based on 100 simulations have a theoretical standard error of 0.050 or less. Five sets of 100 simulations using the unmatched design with five controls produced values having mean 0.460 and standard deviation 0.0474, close to the theoretical standard error 0.0498.

For the rare exposure case studied by simulation, both the matched and counter-matched designs with three or more controls dramatically improved the power of the test for departure from the pure additive model compared with the unmatched design (Table 1). The counter-matched design was clearly superior; it achieved 94 percent power with three controls per case, whereas nine matched controls were required to achieve this level of power. None of the methods was able to detect the subtle departure from a pure multiplicative model, even with nine controls per case, and none of the methods displayed acceptable power against either the multiplicative or additive model with just one or two controls per case. With two controls, counter matching in this situation performed better when the extra control was unexposed rather than exposed.

**Table 1.** Power of the likelihood ratio tests* for interaction.

Case-control ratio	Sampling method		

	Matched	Counter-matched	Unmatched
		*Additive model*	
1:1	0.26	0.36	0.20
1:2	0.39	2 unexposed, 1 exposed: 0.45	0.33
		1 unexposed, 2 exposed: 0.35	
1:3	0.62	0.94	0.38
1:5	0.72	0.94	0.39
1:9	0.95	1.00	0.71

		*Multiplicative model*	
1:1	0.01	0.02	0.06
1:2	0.02	2 unexposed, 1 exposed: 0.00	0.03
		1 unexposed, 2 exposed: 0.02	
1:3	0.00	0.00	0.00
1:5	0.00	0.00	0.01
1:9	0.00	0.00	0.00

* Test size (probability of rejection given the null hypothesis of no interaction) was 0.05.

### Multiplicative interaction parameter

**Figure 1.**
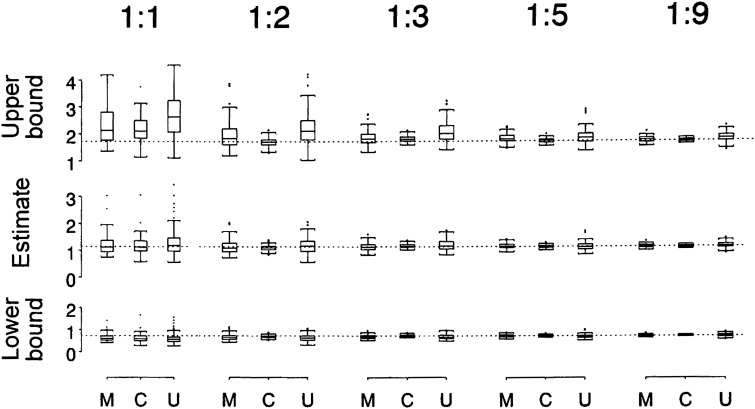
Boxplots exhibiting 100 simulated estimates of the multiplicative interaction parameter *γ*_M_ (equation 2) and 95% likelihood-based confidence bounds for the three sampling methods: matched (M), counter-matched (C), and unmatched (U). Estimates of the parameter and its confidence bounds were obtained by exponentiation of those for *γ*_M_. Results are for case-control ratios of 1:1, 1:2, 1:3, 1:5, and 1:9. Results for counter matching with two controls are from the design with the extra control unexposed. Narrower boxplots reflect smaller sampling variation in the interaction estimates or confidence bounds. More precise estimation of interaction is evidenced by median bounds closer to the interaction estimate. For comparison, the cohort interaction parameter (1.14) and confidence limits (0.74, 1.73) are shown as dotted lines.

The simulated relative efficiencies for the interaction parameter *γ*_M_ are given in Table 2. Estimates and confidence intervals are shown in Figure 1. As with the tests for interaction, both the exposure-matched and counter-matched designs improved efficiency compared to random control selection. The matched and counter-matched designs produced narrower confidence intervals than the unmatched design. Counter-matching produced narrower confidence intervals than those with matching, substantially so in the case of two or three controls. Except for the 1:1 designs, the magnitude of case-control sampling variation in the interaction parameter estimates (standard deviation of simulated estimates) was approximately on the order of 1:2:3 for counter-matched, matched, and unmatched. As a result, counter-matching achieved a level of efficiency — average variance of the interaction estimate — very close to that of the full cohort with five controls (relative efficiency 0.95) whereas matching required nine controls to achieve similar efficiency.

**Table 2.** Small sample (simulated) and asymptotic (calculated) relative efficiencies of the estimates of the log-linear interaction parameter. *

Case-control ratio	Sampling method	Small sample efficiency^†^	Asymptotic relative efficiency^‡^
1:1	Matched	0.42	0.40
	Counter-matched	0.43	0.43
	Unmatched	0.30	0.28

	Matched	0.63	0.58
1:2	Counter-matched:		
	2 unexposed, 1 exposed	0.89	0.80
	1 unexposed, 2 exposed	0.49	0.45
	Unmatched	0.48	0.44

1:3	Matched	0.74	0.68
	Counter-matched	0.90	0.85
	Unmatched	0.59	0.54

1:5	Matched	0.85	0.78
	Counter-matched	0.95	0.91
	Unmatched	0.73	0.66

1:9	Matched	0.95	0.87
	Counter-matched	0.98	0.95
	Unmatched	0.86	0.78

* Relative efficiencies are given for the model with exp{*α*} = exp{*β*} = 2 and exp{*γ*} = (8/7).

^†^ The small sample efficiency is the ratio SE^2^( $\hat{\gamma }$ )_cohort_/SE^2^( $\hat{\gamma }$ )_sample_ of squared standard errors of the parameter estimates from the simulated data.

^‡^ The asymptotic relative efficiency is the ratio SE^2^( $\hat{\gamma }$ )_cohort_/SE^2^( $\hat{\gamma }$ )_sample_ of squared asymptotic standard errors.

### Asymptotic relative efficiency

Results of calculated efficiency corresponding to the parameter values used in the simulations are also given in Table 2; they parallel those based on the simulation and are qualitatively concordant with them. With one control, the matched and counter-matched designs had similar efficiencies that were about 40 percent that of the full cohort, as compared to 0.28 for unmatched. But with three controls, counter-matching was superior. The counter-matching efficiency was 0.85, whereas that for matching was 0.68. With two controls, counter matching was more efficient than matching when the extra control was unexposed (efficiency 0.80), but when the extra control was exposed the efficiency (0.45) was little better than that of the 1:1 design (0.43). The calculations indicate that, in order to obtain the same amount of efficiency as with three counter-matched controls, about eight matched controls, or well over ten unmatched controls, would be required.

**Figure 2.**
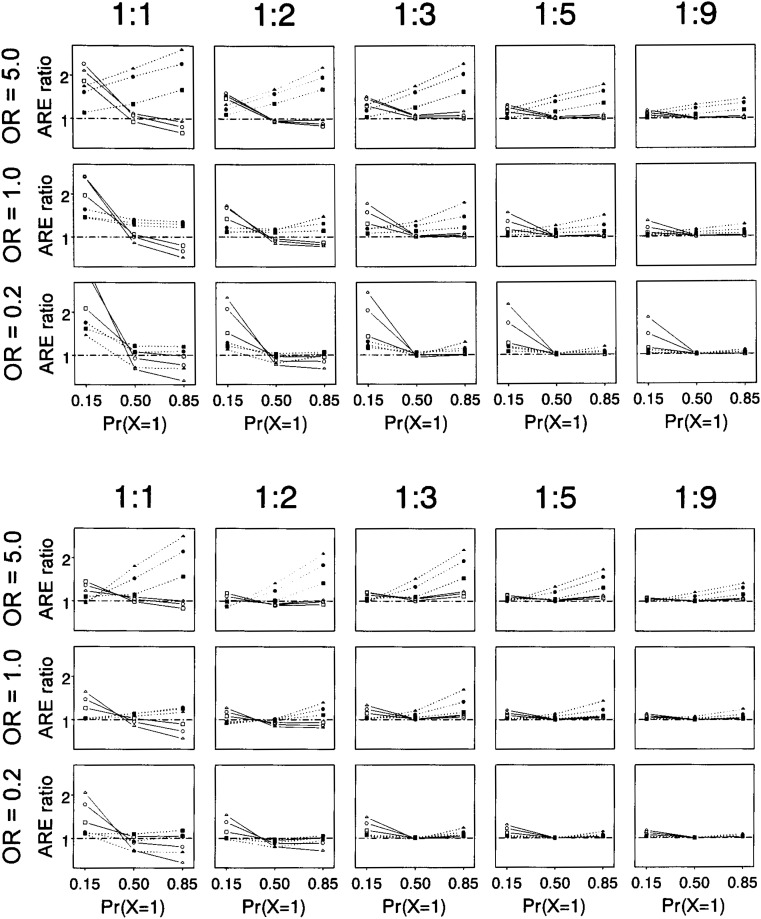
Results of asymptotic relative efficiency (ARE) calculations versus three levels of prevalence of exposure [Pr(*X*=1)]. Points are the ratio of ARE for counter matching to that for matching (▲ ● ■) or random sampling (△ ○ □). Three levels of prevalence of the other factor, *Z*, are compared: Pr(Z=1) = 0.1 (▲ △), Pr(Z=1) = 0.25 (● ○), and Pr(Z=1) = 0.5 (■ □). Relative efficiencies were calculated for three levels of correlation between *X* and *Z*: odds ratio (OR) = 5.0, 1.0, or 0.2. Three values of the interaction between exposure *X* and other factor *Z* were examined: exp{*γ*}=5.0 (upper panel), exp{*γ*}=0.2 (lower panel), and exp{*γ*}=1.0 (not shown; results were intermediate to the other two). Results are for case-control ratios of 1:1, 1:2, 1:3, 1:5, and 1:9. With two controls, the AREs of counter matching for the two allocation strategies were averaged.

The results of the more general asymptotic relative efficiency calculations are shown in Figure 2 for positive (greater-than-multiplicative) interaction (exp{*γ*}=5; upper panel) or negative (less-than-multiplicative) interaction (exp{*γ*}=0.2; lower panel). Results for no interaction — exp{*γ*}=1 — were intermediate to the other two and so are not shown. The result for counter matching with 2 controls is averaged over the two allocation strategies. Efficiency of counter matching is shown relative to that of the other designs. Counter matching was generally more efficient than the other designs (ratio of relative efficiencies often much greater than 1.0) over all of the configurations studied with three or more controls. With rare (prevalence 0.15) *X* and positive interaction, counter matching was the most efficient regardless of number of controls, followed by matching, with the unmatched design having the lowest efficiency. With two controls, the efficiency of counter matching varied depending on whether the extra control was exposed or unexposed. The strategy with the extra control exposed generally performed better, but the strategy with the extra control unexposed had higher efficiency for the combination of rare *X*, rare *Z*, and small interaction (not shown). Large sample relative efficiencies for one control per case were mixed, with counter-matching generally more efficient than the matched or unmatched designs over a wide range of situations, but less efficient than the unmatched design with a common (prevalence 0.85) *X*. Efficiency of the matched design only exceeded that of the unmatched design with a rare (prevalence 0.15) *X*.

## DISCUSSION

It has been demonstrated here and elsewhere that counter matching on a known exposure in nested case-control studies can improve the efficiency of statistical inference about interaction. It is not difficult to intuit why that should be so. If controls are selected randomly with respect to exposure, the resulting sample might be sparse with respect to information about the joint distribution of exposure and the other risk factor, particularly if the exposure is rare. It has further been shown here that counter matching is equal or superior to matching in terms of efficiency. The following three points should therefore be considered by investigators planning nested case-control studies of statistical interaction between two risk factors when one factor is measured in the full cohort:

• Matching complicates exploration of scales of interaction other than those in which the joint effects are multiplicative because the marginal effect of exposure does not factor out of the risk equation and cannot be estimated in an exposure-matched design. Additional assumptions and non-standard methods are therefore needed to fit general risk models in matched designs. Two important issues are 1) possible bias from use of the unadjusted cohort exposure risk estimate, and 2) failure to account for uncertainty in this estimate. Counter matching does not impose any restrictions on the types of general relative risk models that can be explored and standard methods and software may be used.
• Even with standard, multiplicative models, the only situation where matching on a known risk factor might be beneficial for studying interaction in a case-control study is when the matching factor is rare. We have shown that counter matching can be substantially more efficient than matching for estimating the magnitude of interaction in this situation when multiple controls are selected per case. We have also shown that counter-matching is generally more efficient than matched or unmatched designs for studying interaction.
• Implementing counter-matching is no more difficult than matching; in fact, matching can be complicated if done thoughtfully.^18^ In both situations one must create exposure strata and randomly sample appropriate numbers of controls from them. Thus, there seems to be little justification for matching on a known exposure in case-control studies aimed at studying interaction. On the other hand, counter-matching appears to be a generally efficient design for case-control studies of interaction^9^^,^^10^^,^^12^; it has already been demonstrated to improve efficiency in case-control studies aimed at estimating the main effect of exposure after controlling for *Z* as a confounder.^9^

There is a substantial literature on matching in case-control studies.^5^^,^^19^^-^^21^ Matching has been shown to improve efficiency for assessing interaction in situations similar to the breast-cancer case-control study described here, when there is a relatively rare exposure associated with disease and a super-multiplicative interaction.^5^^,^^19^ Intuitively, matching potentially increases efficiency, at least in the multiplicative models, because there are fewer parameters to estimate than in unmatched data and the variation in *X* is avoided. This advantage would seem to dissipate with the use of general risk models and the concomitant need to incorporate, as if it were a known parameter, a possibly biased and inherently random estimate of the main effect of *X*.^2^ Another design — randomized recruitment^22^ — is based on frequency matching and can increase efficiency for studying interaction, but it does not allow the researcher to specify the exact number of controls.

To simplify our simulation and analytic study, a dichotomous exposure indicator was used rather than continuous *X* and we selected controls so that half of the case-control set was in each stratum. With an odd case-control set size (even number of controls), whether the exposed or unexposed stratum should have the greater number of subjects might depend on whether the exposure is rare or not, but must be decided independently of the case exposure status. We found that, with the hypothetical data and rare exposure used in our simulation study, placing the extra control in the unexposed stratum resulted in greater efficiency than placing the extra control in the exposed stratum, but the difference virtually disappeared with greater numbers of controls (4, 6, and so on; results not shown). Thus, the problem of allocating the extra control seems to be limited to studies in which at most two controls can be sampled. On the other hand, placing the extra control in the exposed stratum resulted in greater efficiency in most of the situations studied by asymptotic relative efficiency calculations. There is no intuitive way of guessing the efficiency for testing interaction parameters, so studies require consideration of the design on a case-by-case basis.

In actual application, counter-matched sampling would be based on dose strata as described earlier in the text, so with two controls there would be three strata and no problem of allocating an extra control with odd case-control set sizes. Furthermore, matching would be based on actual doses (thereby producing closer matches), and analyses would use continuous dose. This should increase the efficiency of both exposure-based sampling designs relative to random sampling. With counter matching, the more controls per case, the greater the number of exposure strata used, thus bringing more exposure distribution information to the case-control study.

We have assumed for calculating counter matching weights that each risk set contains only one case. There are two options to handle ties (risk sets having more than one case with the same onset age and date). First, the ties can be randomly broken into separate risk sets for each case and controls counter-matched from the resulting sets; the analysis then proceeds as for individually counter-matched sets. The second is to retain the *k*>1 cases in the risk set and select controls so that there are *k* subjects in each sampling stratum (including the cases). Analysis of this strategy would require the conditional logistic methods described in Langholz and Goldstein.^8^ Because it is computationally simpler, we recommend the first strategy unless there are large numbers of ties.

Interest here has focused on the *X* × *Z* interaction, but in practice investigators may want to study the effect of *Z* in its own right. Thus, there may be conflicting priorities between the need to achieve efficiency in the interaction analysis and efficiency in the analysis of the factor *Z* measured in the case-control sample. If the sole purpose of the case-control study were to evaluate *Z*, there would be no point in matching or counter-matching on *X* if *X* and *Z* were not confounded. If there were confounding, then matching on *X* might be a useful approach to studying the main effect of *Z*. However, one should check for evidence of interaction, and counter-matching allows this while still accommodating control of confounding in the analysis.

## APPENDIX

A hypothetical cohort of 30,000 woman was generated with random binary variables assuming the following proportions: 5 percent with significant radiation exposure (dose > 1 Gray), 25 percent never having been pregnant (nulliparous), and no correlation between radiation dose and pregnancy status. Given the random values of these two factors, breast cancer case status was assigned randomly, also as a binary variable, using a background (unexposed, parous) proportion of 3 percent, relative risks for radiation exposure and nulliparity of 2.0, and a slight positive interaction between radiation and nulliparity on the multiplicative scale (joint relative risk 2 × 2 × 8/7 = 4.57). The resulting proportions in the simulated cohort were: 4.99 percent exposed, 25.46 percent never pregnant, 2.95 percent breast cancer cases in background, relative risk for exposure (among parous women) 1.94, relative risk for nulliparity (among unexposed women) 2.10, and joint relative risk (exposed and nulliparous) 4.64. The multiplicative interaction was therefore 4.64/(1.94 × 2.10) = 1.14. All cases (total number 1,436) were used in the simulations. This hypothetical cohort represents a close approximation to that from which the actual case-control study was sampled (in reality, though, the parity status is not known for the entire cohort).

Controls in a nested case-control study would typically be selected from among the risk set for each case, which comprise all members of the cohort at risk at the time of the case diagnosis. Some controls might appear in multiple sampled risk sets or a case might appear as a control at an earlier time. To simplify the simulations, however, we generated independent risk sets. We sampled from these using random sorting followed by selection of the first *m*_e_^(^*^i^*^)^ exposed or *m*_u_^(^*^i^*^)^ unexposed controls within each set, where *m*_e_^(^*^i^*^)^ + *m*_u_^(^*^i^*^)^ = *m* are the numbers of exposed and unexposed controls called for in the design, given the exposure status of the case *i*. For the unmatched design, *m* controls were sampled randomly without regard to exposure status. The recurrence of some subjects in multiple sampled risk sets further contributes to efficiency in nested case-control studies and the resulting lack of independence among risk sets is accommodated by the conditional likelihood,^10^ but given the large size of the Life Span Study cohort it is unlikely that subjects would be sampled repeatedly in a case-control study of the size described here.
